# Outcome Comparison Between Open and Endovascular Aortic Repair for Retrograde Type A Intramural Hematoma With Intimal Tear in the Descending Thoracic Aorta: A Retrospective Observational Study

**DOI:** 10.3389/fcvm.2021.755214

**Published:** 2021-10-18

**Authors:** Kelvin Jeason Yang, Nai-Hsin Chi, Hsi-Yu Yu, Yih-Sharng Chen, Shoei-Shen Wang, I-Hui Wu

**Affiliations:** ^1^Division of Cardiovascular Surgery, Department of Surgery, National Taiwan University Hospital, College of Medicine, National Taiwan University, Taipei, Taiwan; ^2^Graduate Institute of Clinical Medicine, College of Medicine, National Taiwan University, Taipei, Taiwan; ^3^Department of Surgery, Fu Jen Catholic University Hospital, Fu Jen Catholic University College of Medicine, New Taipei City, Taiwan

**Keywords:** intramural hematoma (IMH), acute aortic syndromes, thoracic endovascular aneurysm repair (TEVAR), aortic dissection, open aortic repair

## Abstract

**Objective:** The optimal treatment modality for retrograde type A intramural hematoma (IMH) remains debatable. This study evaluated and compared surgical outcomes and aortic remodeling after open aortic repair and thoracic endovascular aortic repair (TEVAR) in patients with retrograde type A IMH with a primary intimal tear or ulcer like projection in the descending aorta.

**Methods:** A single center, retrospective observational study was performed on patients with retrograde type A IMH undergoing either open aortic repair and TEVAR. From June 2009 and November 2019, 46 patients with retrograde type A IMH who received either open aortic repair or TEVAR at our institution were reviewed for clinical outcomes, including post-operative mortality/morbidity, re-intervention rate and aortic remodeling.

**Results:** 33 patients underwent open aortic repair and 13 underwent TEVAR. Median age was 68 years (interquartile range [IQR] 15.2 years) and 63 years (IQR 22.5 years) for the open repair group and TEVAR group, respectively. The median duration of follow-up for TEVAR patients was 37.6 months and 40.3 months for open aortic repair. No difference in the 5-year estimated freedom from all-cause mortality (82.1 vs. 87.8%, *p* = 0.34), re-intervention (82.5 vs. 93.8%, *p* = 0.08), and aortic-related mortality (88.9 vs. 90.9%, *p* = 0.88) were observed between the TEVAR and open repair group, respectively; however, the open repair group had a significantly higher 30-day composite morbidity (39.4 vs. 7.7%, *p* = 0.037). All patients from both treatment groups had complete resolution of the IMH in the ascending aorta. With regard to the descending thoracic aorta, TEVAR group had a significantly greater regression in the diameter of the false lumen or IMH thickness when compared to the open repair group [median 14mm (IQR 10.1) vs. 5mm (IQR 9.5), *p* < 0.001].

**Conclusion:** TEVAR and open aortic repair were both effective treatments for retrograde type A IMH, in which no residual ascending aortic IMH was observed during follow-up. TEVAR was also associated with lower post-operative composite morbidities and better descending aortic remodeling. In selected patients with retrograde type A IMH, TEVAR might be a safe, effective alternative treatment modality.

## Introduction

Type A intramural hematoma (IMH) is defined by the presence of hemorrhage within the wall of the ascending aorta (AA) in the absence of an intimal flap or false lumen (FL) (1, 2). Several literatures including the International Registry of Aortic Dissection (IRAD) have reported that early and long-term mortality of IMH are comparable to that of classical aortic dissection (3, 4). Currently, urgent surgical repair is recommended due to the risk of progression into aortic dissection, aneurysm formation and rupture (5, 6). When the primary intimal tear (IT) or ulcer like projection (ULP) are present in the descending thoracic aorta (DTA), it is termed retrograde type A IMH. In contrast to classical type A IMH, retrograde type A IMH may require a more extensive surgical repair with higher surgical mortality and morbidity. As favorable short and midterm outcomes have been reported in patients who underwent Thoracic Endovascular Aortic Repair (TEVAR) for retrograde type A IMH (7–11), it has been increasingly used either alone or in conjunction with open surgical repair (e.g., Frozen elephant trunk (FET) or open antegrade TEVAR) (12–14). Currently, studies reporting the surgical outcomes of both TEVAR and open repair in the treatment of retrograde type A IMH is lacking. Our study aimed to analyze the outcomes in patients with retrograde type A IMH after isolated TEVAR or open repair at our institution.

## Materials and Methods

### Patient Population

**Figure 1:**
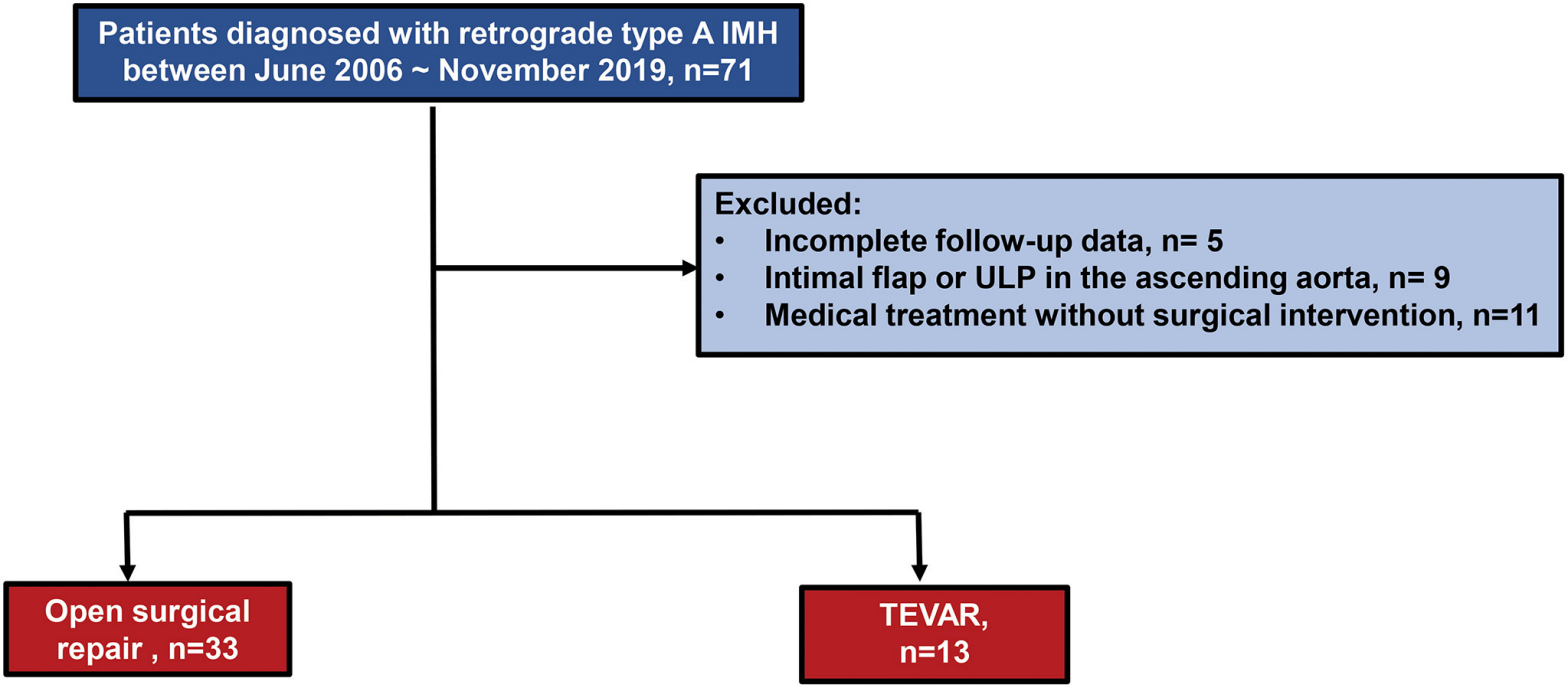
Flowchart of selection of study population.

A diagnostic search from our institutional integrated database was carried out to filter out patients with a diagnosis of aortic dissection or IMH. Individual chart and radiological image review were then performed to identify those with retrograde type A IMH with primary IT or ULP located in the DTA who had received treatment at our institution between June 2009 and November 2019 (Figure 1). Patients with the followings were excluded: (1) classical dissection of the AA having visible intimal flap and communication of the true and FL (2) type A IMH with ULP in the AA.

The diagnoses were established based on specific radiological features observed on contrast-enhanced computed tomography (CT) and transthoracic echocardiography (TTE) or trans-esophageal echocardiography (TEE). The definition of retrograde type A IMH was defined as “the presence of a crescentic or circular high-attenuation area in the AA without contrast enhancement on contrast CT in either the arterial or venous phase, with typical type B aortic dissection (TBAD) or IMH presenting as primary IT or ULP in the DTA” (10). TTE or TEE were used in cases of ambiguity to confirm that no direct flow communication was present within the thickened aortic wall. The decision to receive either TEVAR or open surgical repair was made after a multi-disciplinary meeting involving cardiac surgeons, vascular surgeons and anesthesiologists. The risk and benefit of each surgical option were explained to the patient and family, with patient preference taken into account in the shared decision making process.

Demographics, underlying comorbidities, outcomes and follow-up data were retrieved from our integrated medical database, electronic medical records, and through telephone interview. Measured outcomes included 30-day mortality/composite morbidity, post-operative aortic remodeling and freedom from all-cause mortality/aortic-related mortality/re-intervention. The composite end point of morbidity was defined as the composite of the following: respiratory failure requiring ventilator support for more than 48 h post-operatively, renal failure needing renal replacement therapy, cerebral vascular event, spinal cord ischemia, cardiogenic shock, surgical wound infection and any procedural related adverse events that lead to further intervention. For aortic-related mortality, it was defined as death from either aortic rupture, mal-perfusion, or aortic dissection.

Additionally, pre-operative and post-operative CT images were examined to evaluate the aortic morphology and remodeling of the aortic wall. The aortic remodeling was evaluated by measuring the pre-operative and post-operative maximum aortic diameters of the ascending and descending aorta, IMH thickness of the AA, and FL diameter or IMH thickness in the descending thoracic aorta at the level of pulmonary artery bifurcation in the short axis view of contrast-enhanced CT using the TeraRecon System (TeraRecon Inc., San Mateo, CA, USA).

This study was approved by the institutional review board of National Taiwan University Hospital (NTUH – 202101046RINA) and the board waived the need for informed consent.

### TEVAR

A suitable candidate for TEVAR should fulfill the followings: (1) absence of severe aortic regurgitation (≧grade 3) (2) no evidence of coronary or cerebral ischemia (3) favorable anatomy of the femoral and iliac arteries. The techniques for TEVAR have been described previously (10). In brief, all patients were transferred to the intensive care unit (ICU) for aggressive blood pressure control and pain management after diagnosis. Indications for urgent intervention included persistent chest pain, unstable hemodynamics or pericardial effusion with evidence of tamponade. All TEVAR procedures were carried out under general anesthesia and direct femoral cut-down or percutaneous access within a hybrid operating room. The landing zone, which was based on the Ishimaru classification (15), and stent graft size were selected based on measurements obtained from the pre-operative CT image. Proximal and distal sealing should be of at least 2cm with an oversize of no more than 10% larger than the maximal aortic diameter including the IMH at the proximal landing zone. When there was insufficient landing zone proximal to the ulcer or intimal tear, addition of chimney stents in the supra-aortic branches were used. No post-procedure ballooning was performed. Blood pressure was strictly controlled at ≦130/80mmHg post-operatively in the ICU. No routine spinal drainage was required due to the emergent nature and short segment of DTA coverage.

### Open Aortic Repair

Midline sternotomy was performed and cardiopulmonary bypass (CPB) was commenced after cannulating the right axillary artery, common femoral artery, right atrium and common femoral vein, with systemic cooling down to 25 ~ 28°C. After direct antegrade cardioplegia infusion, moderate hypothermic arrest was initiated and the innominate artery was clamped, followed by antegrade cerebral perfusion via an 8mm side-graft sewn to the right axillary artery and left common carotid artery cannulation (15°C, flow rate of 10–15ml/kg/min). AA/hemiarch/total arch replacement was then performed firstly by completing the distal anastomosis with or without debranching of the supra-aortic vessels. Upon completion of the distal anastomosis, systemic perfusion was reinstituted through the right axillary/femoral artery and rewarming was initiated. The exact extent of the repair was at the discretion of the operating surgeon, where in some patients total arch/hemiarch replacement was performed. In some patients, antegrade TEVAR under direct vision was selectively performed to address the DTA pathology. Lastly, the proximal anastomosis was completed with the use of Teflon felt.

### Follow Up

Post-operatively, follow-up CT image was scheduled at 1 week, 3-months, and 6- months after the intervention and annually thereafter. In order to assess aortic remodeling, the following parameters were measured and compared with the baseline pre-operative images: (1) the maximal diameters of the AA and DTA (2) thickness of the IMH in the AA (3) diameter of the FL or thickness of the IMH in the DTA at the level of pulmonary artery bifurcation. Due to the relatively small case numbers, prolonged study period over 13 years and non-consecutive enrollment with variable follow-up period, only the most recent post-operative CT images were used for analysis. Follow-up clinical data including the 30-day in-hospital mortality, aortic related mortality and long-term survival were obtained from following sources: medical charts, outpatient appointment, telephone interview and the institutional integrated medical database.

### Statistical Analysis

Continuous variables that were normally distributed were displayed as mean ± standard deviation (SD) and analyzed using student *T*-test. Continuous variables with a skewed distribution were displayed as median and 25–75% interquartile range (IQR) and were analyzed using Mann-Whitney test and Kruskal-Wallis test. The Conover test was used as a post-hoc test when the *p*-value for Kruskal-Wallis test was <0.05. Categorical variables were presented as numbers and percentage, and were examined using Fisher's exact test. The all-cause mortality, freedom from aortic related event and freedom from re-intervention were modeled using Kaplan-Meier method, and the log-rank test was applied to test for statistical significance. A *p* < 0.05 was considered statistically significant. All statistical analysis was performed using MedCalc statistical software version 19.5.3 (MedCalc Software, Ostend, Belgium).

## Results

### Demographics

This study included a total of 46 patients with retrograde type A IMH. 13 received TEVAR and 33 received open repair. The demographics and distribution of our cohort for each surgical approach over the study period are displayed in Table 1 and Supplementary Figure 1, respectively. When considering the timing of intervention, 72.7% of those who received open repair were performed within 24 h, in contrast to only 46.2% in the TEVAR group.

**Table 1:** Pre-operative patient characteristics.

**Demographics**	**TEVAR**	**Open**	***p*-value**
	**(*n* = 13)**	**(*n* = 33)**	
Sex, Male (*n*, %)	12 (92.31%)	19 (57.58%)	0.03
Age	63 (22.50)	68 (15.25)	0.25
Pre-operative character (*n*, %)			
Diabetes mellitus	2 (15.38%)	6 (18.18%)	0.82
Dyslipidemia	1 (7.69%)	5 (15.15%)	0.50
Hypertension	11 (84.62%)	25 (75.76%)	0.52
Coronary artery disease	2 (15.38%)	6 (18.18%)	0.82
Prior CABG	1 (7.69%)	1 (3.03%)	0.49
Prior TIA/stroke	2 (15.38%)	2 (6.06%)	0.32
Atrial fibrillation	0 (0%)	2 (6.06%)	0.40
Chronic kidney disease	2 (15.38%)	2 (6.06%)	0.32
AAA s/p EVAR	2 (15.38%)	1 (3.03%)	0.13
Peripheral arterial disease	2 (15.38%)	2 (6.06%)	0.32
COPD	1 (7.69%)	3 (9.09%)	0.88
Liver cirrhosis	1 (7.69%)	0 (0%)	0.11
Surgery <24 h	6 (46.15%)	24 (72.73%)	0.09
Hemopericardium (*n*, %)	4 (30.77%)	14 (42.42%)	0.47
Cardiac tamponade	1 (7.69%)	3 (9.09%)	0.88
Severe aortic insufficiency (≧grade III)	0 (0%)	2 (6.06%)	0.37
Ascending aneurysmal dilatation (≧5 cm)	2 (15.38%)	15 (45.45%)	0.06

*Values are presented as median (IQR) for continuous variables. Categorical variables are presented as n (%). TEVAR, thoracic endovascular aortic repair; CABG, coronary artery bypass grafting; TIA, transient ischemic attack; EVAR, endovascular aortic repair; COPD, chronic obstructive pulmonary disease; IQR, interquartile range*.

### Pre-operative CT Morphology

All patients had CT prior to intervention for the diagnosis and evaluation of aortic morphology (Table 2). For the AA, the open repair group appeared to have a greater pre-operative aortic diameter than the TEVAR group [48.68 ± 6.07 mm vs. 44 ± 5.12 mm]. The IMH thickness was also greater in the open repair group [12.69 ± 4.97mm vs. 7.78 ± 2.42 mm]. Regarding the primary pathology of the DTA, 15 patients presented as TBAD with a primary IT in the DTA, whereas the remaining 31 patients presented as IMH with a ULP in the DTA. Among the 15 patients with a primary pathology of TBAD, eight received TEVAR and seven received open repair. The respective mean DTA diameter and FL diameter were 35.63 ± 4.93 and 19.88 ± 6.31mm for the TEVAR group and 32 ± 1.83 and 12.43 ± 4.96mm for the open repair group. For those presented with IMH with ULP, five had TEVAR and the remaining 26 had open repair. The respective mean DTA diameter and IMH thickness were 36.2 ± 3.03 and 10.52 ± 3.96 for the TEVAR group, 33.85 ± 3.5 and 7.77 ± 4.96 for the open repair group.

**Table 2:** Pre-operative and post-operative CT morphology of the ascending and descending aorta.

**Pre-operative**	**TEVAR**	**Open**	***p*-value**
**CT morphology**	**(*n* = 13)**	**(*n* = 33)**	
Ascending aorta			
Aortic diameter	44 ± 5.12	48.68 ± 6.07	0.03
IMH thickness	7.78 ± 2.42	12.69 ± 4.97	0.002
Descending aorta			
*Dissection*	*n* = 8	*n* = 7	
Aortic diameter	35.63 ± 4.93	32 ± 1.83	0.08
False lumen diameter	19.88 ± 6.31	12.43 ± 4.96	0.02
Tear location^*^	13 (10)	20 (13.5)	0.40
*IMH*	*n* = 5	*n* = 26	
Aortic diameter	36.2 ± 3.03	33.85 ± 3.50	0.18
IMH thickness	10.52 ± 3.69	7.77 ± 4.96	0.17
PAU or ULP location^*^	30.0 (75.0)	0 (10.0)	0.01
**Post-operative CT morphology**			
Ascending aorta			
Diameter	39.69 ± 6.05	34.26 ± 2.67	0.002
IMH thickness	0 (0)	0 (0)	0.15
Descending aorta			
Diameter	31.46 ± 5.24	32.15 ± 4.73	0.43
FL diameter or IMH thickness	0 (0)	0 (90.0)	0.12

*All diameters / thickness / length are measured in millimeters (mm)*.

*For pre-operative CT morphology of the ascending aorta, aortic diameter and IMH thickness were displayed. For the descending aorta, the lesions were grouped depending on whether it is an intimal tear or ulcer-like projection. Values are presented as mean ± SD or median (IQR) for continuous variables. Categorical variables are presented as n (%). CT, computed tomography; TEVAR, thoracic endovascular aortic repair; IMH, intramural hematoma; SD, standard deviation; IQR, interquartile range; PAU, penetrating aortic ulcer; ULP, ulcer like projection; FL false lumen*.

* *Millimeters distal to the left subclavian artery*.

### Operative Details

#### TEVAR

The procedural details for TEVAR were displayed in Supplementary Table 1. Stent grafts were successfully deployed in all 13 patients with complete coverage of the IT and ULP in the DTA. The median length of coverage was 15cm (IQR 5.0). One patient (7.7%) had proximal landing in zone 1, three (23.1%) in zone 2, eight (61.5%) in zone 3, and one (7.7%) in zone 4. Chimney stents were required in four patients who had their proximal landing in zone 1 and zone 2, including 3 in the left subclavian artery and one in the left common carotid artery. Two patients also underwent pericardiocentesis for the treatment of pericardial effusion. 46.2% of our patients had the TEVAR stent implanted within 24 h of presentation. Re-intervention for distal intimal tear was required in two (15.4%) of the 13 patients and the time to re-intervention were at nine and 16 months, respectively.

#### Open Repair

The operative details for open repair were shown in Supplementary Table 1. A total of 33 patients received open repair for retrograde type A IMH. Among them, 16 underwent AA grafting, 15 underwent hemiarch replacement with open antegrade TEVAR and two underwent total arch replacement with frozen elephant trunk (FET). The median CPB time, cross-clamp time and cerebral perfusion time were 209 min (IQR 55.5), 123 min (IQR 52.0), and 58.5 min (IQR 43.0), respectively. In four of our patients, additional procedures were performed, which included three aortic valvuloplasty, one coronary artery bypass grafting and one veno-arterial extracorporeal membrane oxygenation.

### Post-operative Outcomes

**Figure 2:**
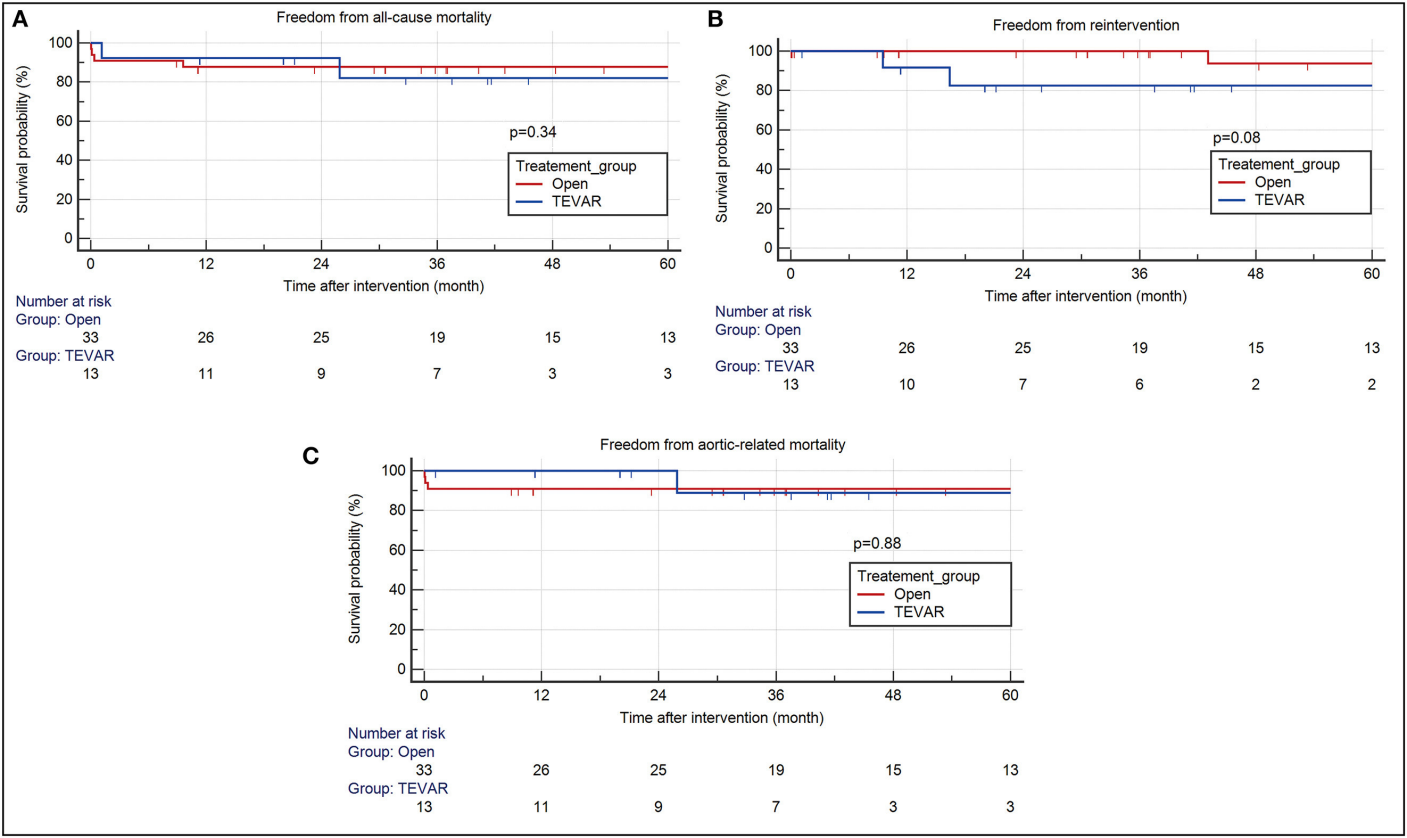
Kaplan Meier survival analysis of patients after TEVAR and open aortic repair. There were no differences between the two intervention group in terms of **(A)** all-cause mortality (TEVAR- HR 2.28, 95% CI 0.42–12.24; open- HR 0.44, 95% CI 0.082–2.36) **(B)** re-intervention (TEVAR- HR 10.51, 95% CI 0.73–15.1; open- HR 0.1, 95% CI 0.07–1.37) **(C)** aortic related mortality (TEVAR- HR 0.85, 95% CI 0.1–7.49; Open – HR 1.18, 95% CI 0.13–10.37) at 60-month follow-up. TEVAR, thoracic endovascular aortic repair; HR, hazard ratio; CI, confidence interval.

The median follow-up duration was 37.57 months (IQR 29.75) for the TEVAR group and 40.33 months (IQR 67.74) for the open repair group. 30-day composite morbidity was higher in patients after open repair, in comparison to TEVAR [13/33 (39.39%) vs. 1/13 (7.69%)]. Stroke, post-operative bleeding requiring re-exploration, renal failure and myocardial stunning were the most common comorbidities after open repair (Table 3). 30-day mortality occurred in three patients after open repair due to multi-organ failure. Though none occurred within 30 days after TEVAR, there was one liver cirrhotic patient who died on post-operative day 34 due to subdural hemorrhage, which was considered unrelated to the TEVAR procedure or primary aortic pathology. The median operation time was shorter in patients who underwent TEVAR [125 min (IQR 44.5) vs. 373 min (IQR 145.5)]. Two patients (15.4%) required re-intervention after TEVAR and the timing for re-intervention were at 9 months and 16 months, respectively. One patient had a distal stent-graft induced new entry tear (SINE) and the other patient had a newly developed penetrating aortic ulcer (PAU) that was located 9 cm distal to the old stent-graft. Subsequent intervention of the DTA was required in one patient after open repair because of the development of new PAU in the DTA, for which the patient had received TEVAR. There was an increase in re-intervention rate in the TEVAR group (15.38%) in comparison to open repair (3.03%), though the difference was statistically insignificant. On survival analysis, no difference in the 5-year estimated freedom from all-cause mortality (82.1 vs. 87.8%, *p* = 0.34), re-intervention (82.5 vs. 93.8%, *p* = 0.08), and aortic-related mortality (88.9 vs. 90.9%, *p* = 0.88) were observed between the TEVAR and open repair group, respectively (Figure 2).

**Table 3:** Post-operative outcomes.

**Outcomes**	**TEVAR**	**Open**	***p*-value**
	**(*n* = 13)**	**(*n* = 33)**	
Follow-up duration (month)	37.57 (29.75)	40.33 (67.74)	0.40
30-day mortality (*n*, %)	0	3 (9.09%)	0.27
30-day morbidity (*n*, %)	1 (7.69%)	13 (39.39%)	0.04
Morbidity types (*n*, %)			
Cerebral vascular event	1 (7.69%)	7 (21.21%)	0.28
Spinal cord infarction	0 (0%)	1 (3.03%)	0.53
Cardiogenic shock	0 (0%)	3 (9.09%)	0.27
Respiratory failure	0 (0%)	2 (6.06%)	0.37
Permanent pacemaker	n/a	1 (3.03%)	0.53
Sternal wound infection	n/a	2 (6.06%)	0.37
Deep vein thrombosis	0 (0%)	1 (3.03%)	0.53
Re-exploration	n/a	5 (15.15%)	0.14
Renal failure	0 (0%)	3 (9.09%)	0.27
Operation time (min)	125 (44.50)	373 (145.5)	<0.001
Re-intervention (*n*, %)	2 (15.38%)	1 (3.03%)	0.13

*This table shows the respective surgical outcomes for patients with retrograde type A intramural hematoma treated with TEVAR and open repair. Values are presented as median (IQR) or n (%). TEVAR, thoracic endovascular aortic repair; IQR, interquartile range*.

### Aortic Remodeling

**Figure 3:**
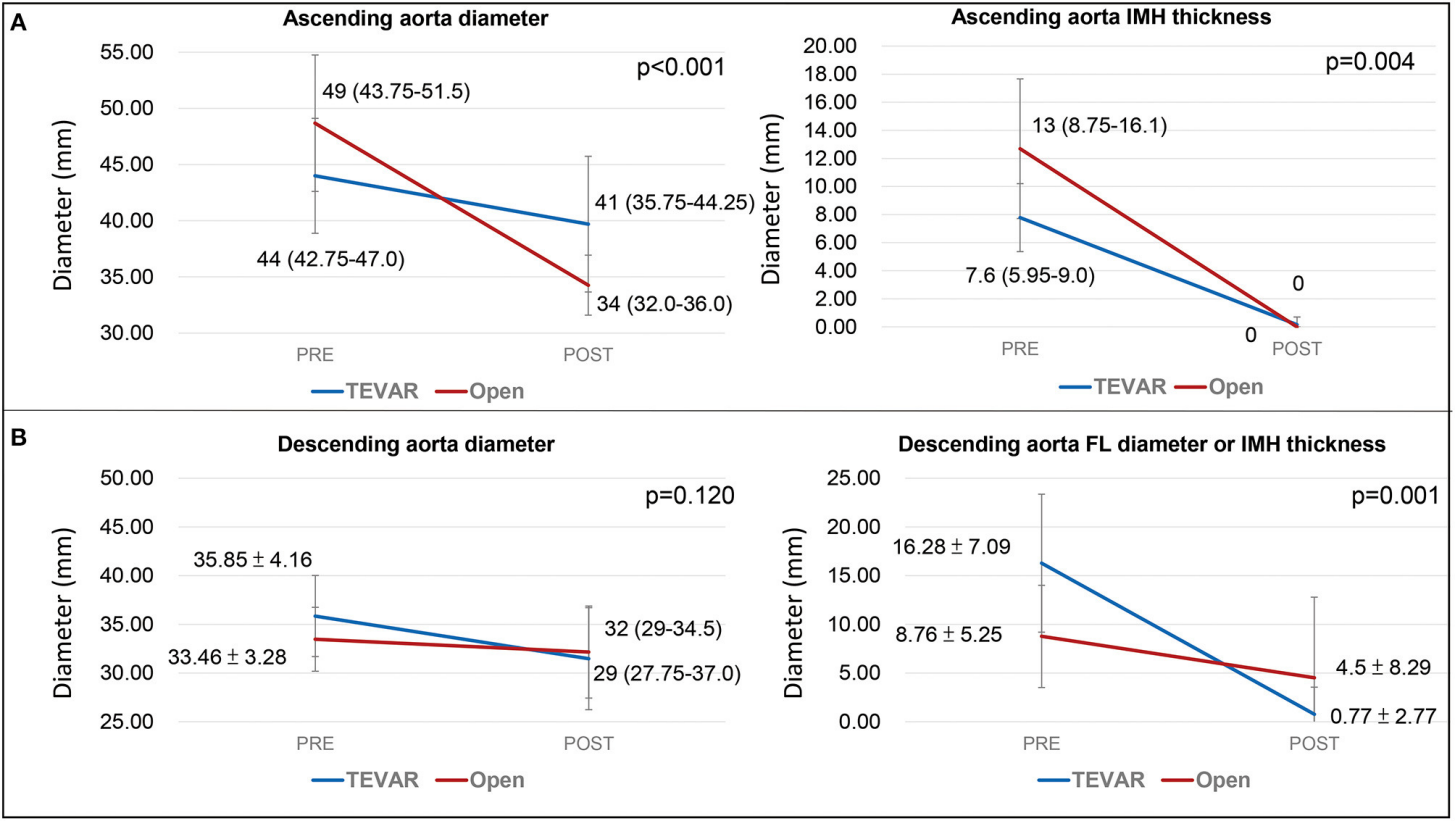
Aortic remodeling of the ascending and descending aorta. **(A)** illustrates the change in aortic diameter and IMH thickness of the ascending aorta on post-operative follow-up. **(B)** shows the change in aortic diameter and IMH thickness/FL diameter post-operatively. The values represents mean diameter ± SD. TEVAR, thoracic endovascular aortic repair; SD, standard deviation; FL, false lumen; IMH, intramural hematoma.

**Figure 4:**
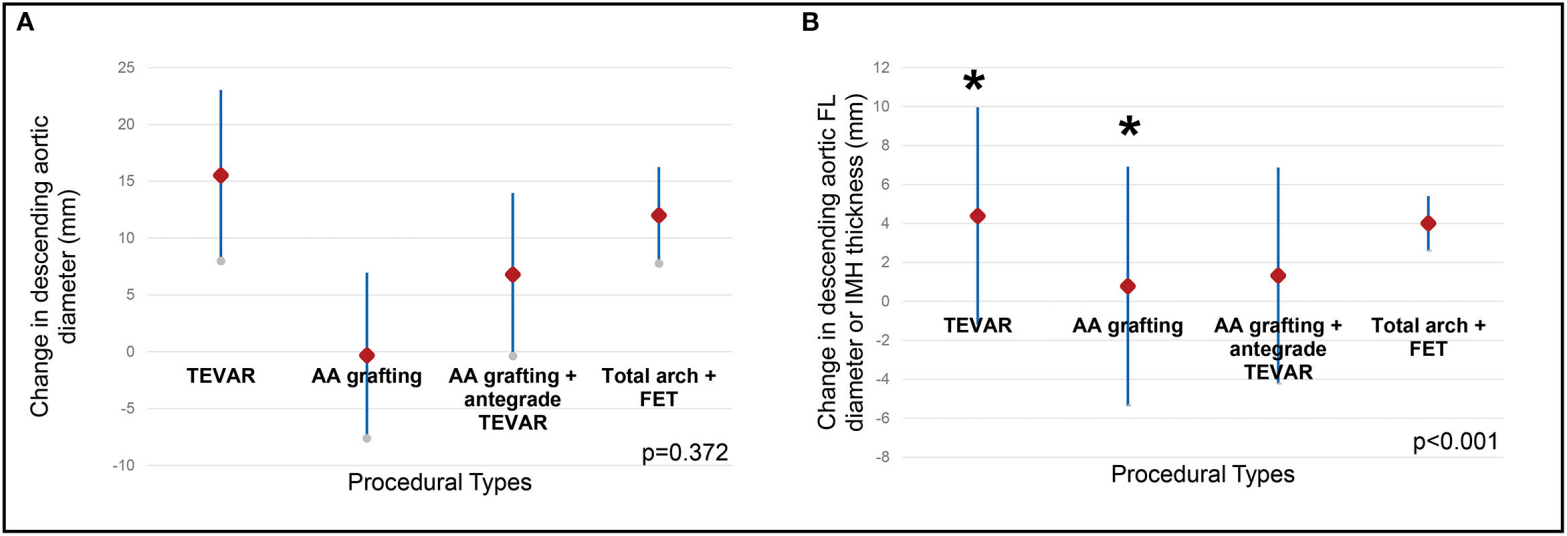
Remodeling of the descending aorta by procedural types. Different types of open procedures were compared with TEVAR using Kruskal-Wallis test. **(A)** the change in descending aortic diameter was comparable between different procedures. **(B)** the change in FL diameter/IMH thickness in the descending aorta was different between procedures (*p* < 0.001), post-hoc analysis revealed the difference was between TEVAR and ascending aortic grafting (indicated by asterisks). TEVAR, thoracic endovascular aortic repair; FL, false lumen; IMH, intramural hematoma; AA, grafting ascending aortic grafting; FET, frozen elephant trunk.

The post-operative CT parameters of the aorta were also displayed in Table 2. Complete resolution of IMH in the AA was observed in all patients after both open repair and TEVAR on follow-up CT. The mean change in AA diameter and IMH thickness were 14.26 ± 5.43 mm / 12.14 ± 4.59mm after open repair, and 4.31 ± 4.29mm / 7.62 ± 2.68mm after TEVAR. For the DTA, no difference was observed between the two treatment modalities in terms of reduction in DTA diameter. However, patients who received TEVAR had a significantly greater regression in the diameter of their FL or IMH thickness when compared to open repair (Figure 3). When different techniques of open repair were analyzed for DTA remodeling (Supplementary Table 2), post-hoc analysis showed a significant reduction in FL diameter / IMH thickness in the DTA after TEVAR compared to AA grafting (Figure 4). However, no difference was observed between TEVAR, total arch replacement with FET and hemiarch replacement with open antegrade TEVAR.

## Discussion

In this study, TEVAR and open aortic repair were both effective in promoting AA remodeling, and TEVAR was associated with lower post-operative composite morbidity and better remodeling of the DTA.

A recently published multi-center study had demonstrated that TEVAR is a safe alternative to open repair, with no 30-day mortality and a low re-intervention rate of only 6%. Post-operative neurological complications developed in two (11.1%) patients and renal/respiratory failure in one (5.6%) (10), and these numbers were substantially lower than open repair, which were reported to be around 12.9%~31.7% and 9.9% ~ 50.5%, respectively (4, 16–18). In our study, the short-term composite morbidities were significantly higher for patients after open repair, consisting of post-operative stroke, renal failure and bleeding etc. In addition, three patients died within 30-days after open repair due to multi-organ failure, whereas TEVAR had no 30-day mortality except one cirrhotic patient who died on post-operative day 34 because of subdural hemorrhage that was unrelated to the procedure or primary aortic pathology.

Although more re-interventions were required after TEVAR repair within 3-year of follow-up, these procedures were all simple TEVAR extension in the distal thoracic aorta for distal SINE. Neither retrograde type A aortic dissection nor late aortic related deaths was observed. In the previous report, it was emphasized that patient selection and pre-operative surgical planning were all crucial for the success of TEVAR (10). Important aspects of the surgical techniques include the followings: (1) Ensure no detectable flow is present in the ascending IMH (2) Oversize of the stent graft no more than 10% at the proximal landing zone. (3) Avoid post-procedural aortic ballooning (4) Strict post-operative blood pressure control.

For aortic remodeling, both open repair and TEVAR could promote AA remodeling by reducing the AA diameter, as well as achieving complete resolution of the IMH. Evidence gathered from studies of TBAD have demonstrated that aortic remodeling was a positive prognostic factor after TEVAR, and was associated with a reduced risk of aortic related complications (e.g., aortic rupture, aneurysmal dilatation), and might therefore improve long-term survival (19–21). According to most Western guidelines, open repair remained the standard treatment for type A IMH, particularly for those with hemodynamic instability, impending rupture or persistent symptoms (5, 6, 22–25). In contrast, some East Asian centers have adopted a more conservative approach with initial medical treatment in relatively stable patients. However, the 5-year aortic event free survival was as low as 52.7%, and the rates of in-hospital mortality/conversion to surgery in these patients ranged from 4–7% and 20–35%, respectively (26–32). In this study, the intended benefit of TEVAR was to seal the primary IT tear in the DTA with a single, endovascular procedure. Whereas, in open repair, a more aggressive approach was usually mandated for the distal tear. In our TEVAR cohort, the in-hospital mortality was 0% and we have not observed any event of reverse aortic remodeling or dissection of AA after a median follow-up of 3-year. The primary IT closure could depressurize the IMH in the AA and promote AA remodeling (7–11). For DTA remodeling, significant better aortic remodeling was observed in the TEVAR repair compared to open repair, especially with AA grafting only. This is unsurprising as TEVAR directly addresses the DTA pathology.

With regards to the impact of follow-up duration on the difference in aortic remodeling, the median follow-up duration for TEVAR and open repair were 37.5 and 40.3 months, respectively. All patients from both treatment groups had complete resolution of the IMH in the AA, and resolution all occurred within 1 year after TEVAR. However, TEVAR patients were at risk stent graft induced new entry tear and progression of intrinsic descending pathology. Studies have also shown a 10–40% risk of late reoperation for patients undergoing open repair (33, 34). Therefore, we believe long-term CT follow-up is still mandatory for both open repair and TEVAR.

Our findings were consistent with previous studies (10), suggesting that TEVAR is effective in promoting aortic remodeling, in both the AA and DTA. When subgroups within the open repair were compared, the remodeling of the DTA was better in patients who had received either FET or open antegrade TEVAR during open repair, whereas in those who received isolated AA grafting showed progressive enlargement of the DTA during follow-up.

### Study Limitation

Our study was a single center retrospective study with a relatively small case number and non-consecutive case enrollment. The study cohort was heterogeneous in nature, which might have impact on data analysis and interpretation. Though general consensus on the indication for TEVAR or surgical aortic repair were in place, the ultimate decision remained at the discretion of the operating surgeon, after discussing the potential pros and cons with the patient. This might result in selection bias. The presence of selection bias was suggested by the following: (1) 72.7% of those who underwent open repair were operated within 24 h, compared to only 46.2% for the TEVAR cohort (2) More patients in the open repair group had severe aortic insufficiency and aneurysmal dilatation of the aorta prior to intervention (3) 72.7% of those who received open repair had an IMH thickness >11mm & ascending aortic diameter >50mm, in contrast to only 23.1% in the TEVAR group.

Currently, the standard treatment for type A IMH based on existing guidelines is still open aortic repair. Though TEVAR has been reported by literatures with promising results in the treatment of retrograde type A IMH, it has not been regarded as a standard treatment modality for this particular patient group and the optimal treatment remains controversial among open surgical grafting, TEVAR or medical treatment. Therefore, a validated treatment algorithm to determine which patient should undergo TEVAR is currently lacking and it was one of the reason why this study was conducted. Currently, an ascending aortic IMH thickness > 11mm and a maximum ascending aortic diameter >50mm are in preference to open aortic repair, though the final decision may be greatly affected by factors including patient and physician preference. Further prospective studies with a larger cohort and longer follow-up are necessary in order to draw a firm conclusion on the indication and optimal treatment modality for retrograde type A IMH.

## Conclusions

This was the first study presenting outcome data on both TEVAR and open aortic repair in patients with retrograde type A IMH. In this study, TEVAR and open aortic repair were both effective treatment for retrograde type A IMH. TEVAR promoted remodeling of the AA with complete resolution of the IMH, whereas open aortic repair replaced the diseased AA entirely. From our results, TEVAR might also have the potential advantage of lower post-operative morbidity and better remodeling of the DTA. This study suggested that TEVAR might be a safe, effective alternative treatment modality in selected patients with retrograde type A IMH.

## Data Availability Statement

The raw data supporting the conclusions of this article will be made available by the authors, without undue reservation.

## Ethics Statement

The studies involving human participants this study was approved by the institutional review board of National Taiwan University Hospital (NTUH–202101046RINA) and the board waived the need for informed consent. Written informed consent for participation was not required for this study in accordance with the national legislation and the institutional requirements.

## Author Contributions

KY: data curation and writing—original draft. N-HC: data curation, and review and editing. H-YY: data curation. Y-SC: writing—review and editing. S-SW: conceptualization and supervision. I-HW: conceptualization, methodology, writing—review and editing, validation, and supervision. All authors contributed to the article and approved the submitted version.

## Conflict of Interest

The authors declare that the research was conducted in the absence of any commercial or financial relationships that could be construed as a potential conflict of interest.

## Publisher's Note

All claims expressed in this article are solely those of the authors and do not necessarily represent those of their affiliated organizations, or those of the publisher, the editors and the reviewers. Any product that may be evaluated in this article, or claim that may be made by its manufacturer, is not guaranteed or endorsed by the publisher.

## Supplementary Material

Supplementary Figure 1 Case distribution for each surgical approach during the study period.

## Abbreviations

AA: ascending aorta
DTA: descending thoracic aorta
FL: False lumen
TBAD: type B aortic dissection
IMH: intramural hematoma
IT: intimal tear
ULP: ulcer-like projection
TEVAR: thoracic endovascular aortic repair
FET: frozen elephant trunk
CT: computed tomography
TTE: transthoracic echocardiography
TEE: trans-esophageal echocardiography
CPB: cardiopulmonary bypass
SD: standard deviation
IQR: inter-quartile range
SINE: stent-graft induced intimal tear
PAU: penetrating aortic ulcer.
