# Supplementary material for: Outcome Comparison Between Open and Endovascular Aortic Repair for Retrograde Type A Intramural Hematoma With Intimal Tear in the Descending Thoracic Aorta: A Retrospective Observational Study
Source: Front Cardiovasc Med. 2021 Oct 18;8:755214. doi: 10.3389/fcvm.2021.755214 (PMC8558361; doi:10.3389/fcvm.2021.755214)
Supplement: Supplementary file 2 [file Data_Sheet_1.docx]

**Supplementary Table S1. Perioperative details.**

| **TEVAR group (n=13)** |  |
| --- | --- |
| Device |  |
| Gore C-Tag | 10 (76.9%) |
| Medtronic Valiant  Cook | 2 (15.4%)  1 (7.7%) |
| Proximal landing zone |  |
| Zone 1 | 1 (7.7%) |
| Zone 2 | 3 (23.1%) |
| Zone 3 | 8 (61.5%) |
| Zone 4 | 1 (7.7%) |
| Chimney stent |  |
| LSCA | 3 (23.1%) |
| LCCA + LSCA embolization | 1 (7.7%) |
| Length of coverage (cm) | 15 (5.0) |
| Concomitant procedure |  |
| Pericardial drainage | 2 (15.4%) |
| **Open repair (n=33)** |  |
| CPB time (min) | 209.0 (55.5) |
| Cross-clamp time (min) | 123.0 (52.0) |
| Antegrade cerebral perfusion (min) | 58.5 (43.0) |
| Core temp (℃) | 25 (7.5) |
| Cannulation  Axillary + femoral | 33 (100%) |
| Techniques  Ascending aortic grafting  Total arch with FET  Ascending aortic grafting with antegrade TEVAR | 16 (48.5%)  2 (6.1%)  15 (45.5%) |
| Associated procedure  Aortic valvuloplasty  CABG  ECMO | 3 (9.1%)  1 (3.0%)  1 (3.0%) |

Procedural details of the 13 patients treated by TEVAR and the 33 patients treated by open repair for retrograde type A intramural hematoma. Values are presented as median (IQR) for continuous variables. Categorical variables are presented as n (%). *TEVAR* thoracic endovascular aortic repair, *LSCA* left subclavian artery, *LCCA* left common carotid artery, *IQR* interquartile range, *CPB* cardiopulmonary bypass, *FET* frozen elephant trunk, *CABG* coronary artery bypass grafting, *ECMO* extracorporeal membrane oxygenation

**Supplementary Table S2. Comparison of descending aortic remodeling between different open aortic repair techniques**.

| **Post-operative CT morphology** | Ascending grafting, n=16 | Total arch with FET, n=2 | Hemiarch + antegrade TEVAR, n=15 | TEVAR, n=13 | *p*-value |
| --- | --- | --- | --- | --- | --- |
| Descending aortic diameter | 32.0 (7.15) | 29.0 (2.0) | 31.0 (3.0) | 29.0 (9.25) | .53 |
| Change in diameter | 2.0 (7.15) | 4.0 (2.0) | 2.0 (4.0) | 4.0 (7.5) | **.38** |
| Descending FL diameter or IMH thickness | 0 (14.5) | 0 (0) | 0 (0) | 0 (0) | .15 |
| Change in diameter | **1.5 (11.0)*** | 12.0 (6.0) | 6.5 (7.0) | **14.0 (10.1)*** | **<.001** |
| _All diameters / thickness are measured in millimeters (mm)_ | | | | | |

Values are presented as median (IQR) for continuous variables. Categorical variables are presented as n (%). *TEVAR* thoracic endovascular aortic repair, *IQR* interquartile range, *FL* false lumen, *IMH* intramural hematoma.
