# Supplementary material for: Outcome Comparison Between Open and Endovascular Aortic Repair for Retrograde Type A Intramural Hematoma With Intimal Tear in the Descending Thoracic Aorta: A Retrospective Observational Study
Source: Front Cardiovasc Med. 2021 Oct 18;8:755214. doi: 10.3389/fcvm.2021.755214 (PMC8558361; doi:10.3389/fcvm.2021.755214)
Supplement: Supplementary file 3 [file Presentation_1.PPTX]

## Slide 1
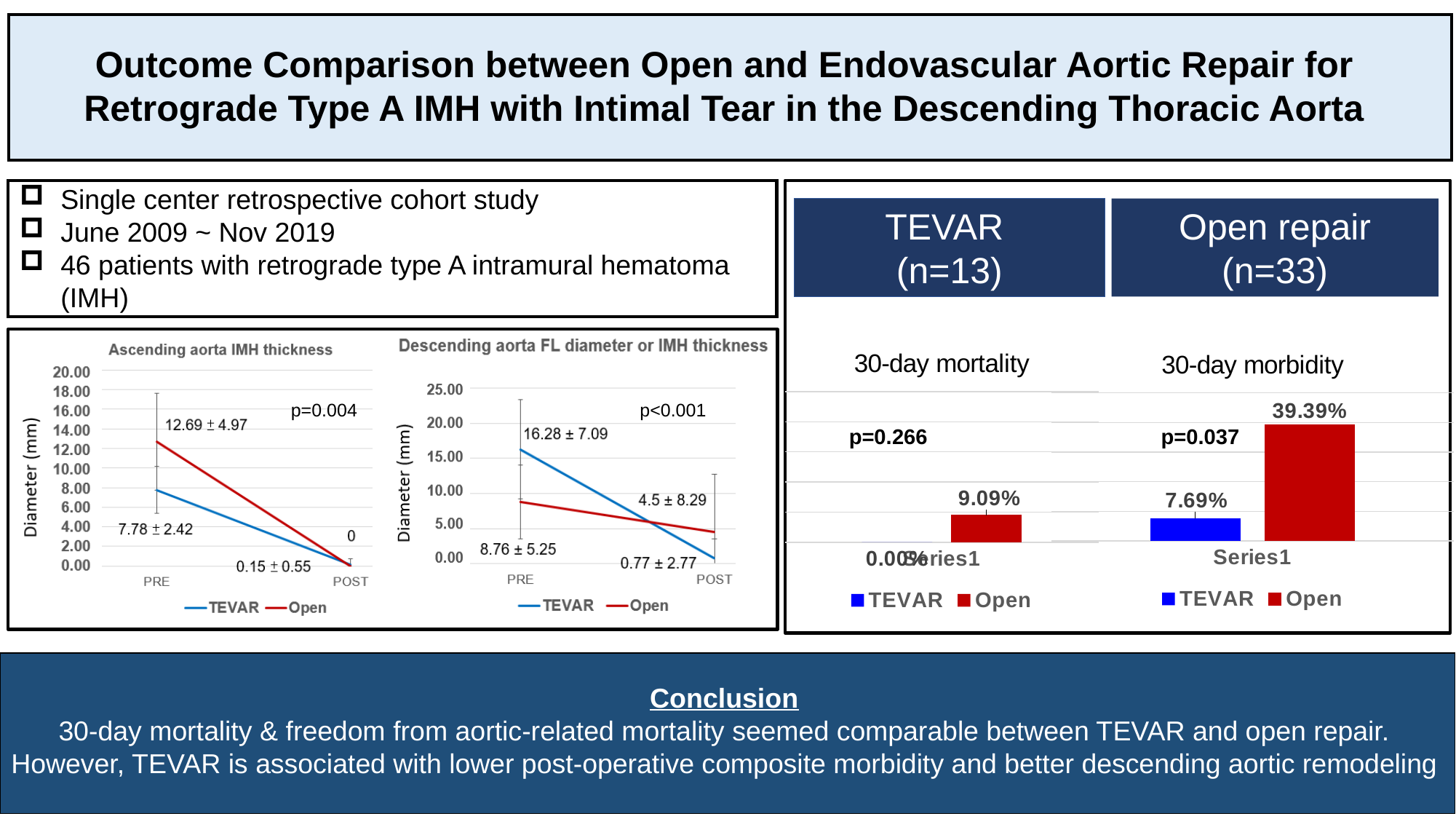

Outcome Comparison between Open and Endovascular Aortic Repair for Retrograde Type A IMH with Intimal Tear in the Descending Thoracic Aorta
Single center retrospective cohort study
June 2009 ~ Nov 2019
46 patients with retrograde type A intramural hematoma (IMH)
TEVAR
(n=13)
Open repair
(n=33)
### Chart: 30-day mortality
| Category | TEVAR | Open |
|---|---|---|
| | 0.0 | 0.0909 |
### Chart: 30-day morbidity
| Category | TEVAR | Open |
|---|---|---|
| | 0.0769 | 0.3939 |
p=0.004
p<0.001
p=0.266
p=0.037
Conclusion
30-day mortality & freedom from aortic-related mortality seemed comparable between TEVAR and open repair. However, TEVAR is associated with lower post-operative composite morbidity and better descending aortic remodeling
